# Office-Based LA-BET Without Sedation or Nerve Block: Prospective Evaluation of a Simplified Local Anesthesia Protocol

**DOI:** 10.3390/jcm15020543

**Published:** 2026-01-09

**Authors:** Cheng-Yu Hsieh, Yi-Fan Chou, Chuan-Jen Hsu

**Affiliations:** 1 Department of Otolaryngology, Head and Neck Surgery, Taichung Tzu Chi Hospital, Buddhist Tzu Chi Medical Foundation, Taichung 427, Taiwan; 2 School of Medicine, Tzu Chi University, Hualien 970, Taiwan; 3 Institute of Clinical Medicine, National Taiwan University College of Medicine, Taipei 100, Taiwan

**Keywords:** eustachian tube dysfunction, balloon eustachian tuboplasty, local anesthesia

## Abstract

**Background/Objectives**: Balloon eustachian tuboplasty (BET) is an effective surgical option for obstructive eustachian tube dysfunction (OETD). However, the feasibility of performing BET under local anesthesia (LA) using simplified analgesic protocols remains underexplored. We examined the feasibility of a streamlined LA-BET protocol. **Methods**: Fifty patients (sixty-four ears) diagnosed with primary OETD between March 2024 and December 2025 were enrolled. All patients underwent BET under LA using intramuscular ketorolac and topical lidocaine gel without sedation or nerve blocks. Pain scores, blood pressure changes, and patient acceptance were analyzed for each patient; Eustachian Tube Dysfunction Questionnaire-7 (ETDQ-7) scores, tympanogram types, and Valsalva results were analyzed for each ear. All outcome measures were assessed 3 months postoperatively. **Results**: The mean ETDQ-7 score significantly improved from 24.9 ± 7.4 to 11.9 ± 5.4 (*p* < 0.001). The minimal clinically important difference (MCID ≥ 3.7) was achieved in 90.6% of ears, and normalization (ETDQ-7 ≤ 14.5) in 75.0%. The proportion of ears with positive Valsalva maneuvers increased from 39.1 to 76.6% (*p* < 0.01), and type A tympanograms improved from 64.1 to 84.4% (*p* = 0.018). Mean pain scores were 3.5 during insertion, 2.1 during balloon inflation, and 0.6 after deflation. All patients completed the procedure, and 96% would undergo LA again. **Conclusions**: LA-BET performed using intramuscular ketorolac and topical lidocaine gel is safe, tolerable, and effective. This protocol provides symptom relief and functional improvement without sedation or nerve block and offers a practical outpatient alternative for chronic OETD management.

## 1. Introduction

Eustachian tube dysfunction (ETD) is commonly classified into obstructive (dilatory), patulous, and baro-challenge types. Among these, obstructive ETD (OETD) is the most prevalent [1] and typically results from mucosal inflammation secondary to otitis media, chronic rhinitis, rhinosinusitis, upper respiratory tract infection, or gastroesophageal reflux [2,3,4]. Patients often present with aural fullness, otalgia, muffled hearing, and a sensation of persistent pressure [1]. Impaired ventilatory and clearance functions can lead to negative middle ear pressure, causing effusion, tympanic membrane retraction, and recurrent otitis media, and creating a substantial burden on adult and pediatric populations. The prevalence of ETD is 5.44% in the adult population and 9.04% among patients with cancer in the USA [5], resulting in millions of healthcare visits each year and underscoring its substantial clinical and public health impact.

Balloon eustachian tuboplasty (BET) has recently become an effective surgical treatment for chronic OETD [6,7]. The inflamed epithelium is crushed by mechanical dilation of the cartilaginous portion of the eustachian tube. BET helps restore physiological ventilation and mucociliary clearance in the middle ear [8,9]. Multiple studies have demonstrated its clinical efficacy in patients with primary OETD, chronic otitis media, and adhesive otitis media, showing improvements in subjective symptoms and objective outcomes [7,10,11,12].

BET is traditionally performed under general anesthesia (GA) to ensure complete patient immobility and minimize discomfort during dilation. However, the procedure is remarkably brief: balloon inflation typically lasts 2–4 min per side and the entire procedure can be completed within 10–15 min. In contrast, GA prolongs operative and recovery times, increases the risk of airway or cardiovascular complications, and incurs considerable costs. In actual clinical practice, many patients hesitate regarding the need for GA because the procedure is so short. Patients may also experience throat pain, nausea, and dizziness postoperatively. Therefore, performing BET under local anesthesia (LA) in an in-office setting may enhance procedural efficiency, reduce healthcare costs, and improve patient convenience.

Early feasibility studies have shown promising results for BET performed under LA. Luukkainen et al. (2017) demonstrated that BET can be safely performed under monitored anesthesia care [13], whereas Dean (2019) and Chen et al. (2020) confirmed its practicality in in-office settings, achieving >90% completion rates and mean visual analogue scale (VAS) scores of 5–6 during dilation [14,15]. More recent large-scale studies, including those by Toivonen et al. (2022) and Møller (2025), found that outcomes achieved under LA were comparable to those achieved under GA, with faster recovery, lower cost, and few complications [16,17]. In Møller’s study, the refinement of the anesthesia protocol markedly reduced pain scores.

Most published LA-BET protocols still rely on sedation, intravenous analgesics, or sphenopalatine ganglion blocks, and a simplified, sedation-free approach has not yet been evaluated. Establishment of a protocol that is effective and well-tolerated is essential to ensure patient comfort and procedural safety. Therefore, in this study we aimed to examine the feasibility, pain tolerance, safety, and efficacy of a streamlined LA-BET protocol to determine its suitability for routine clinical use under LA.

## 2. Materials and Methods

### 2.1. Ethical Considerations

This prospective study was approved by the Research Ethics Committee (REC113-16) and adhered to the guidelines of the Declaration of Helsinki. Written informed consent was obtained from all enrolled patients, following a protocol approved by the ethics committee.

### 2.2. Experimental Design

Fifty patients diagnosed with primary OETD between May 2024 and June 2025 were enrolled in this study. OETD was diagnosed based on the following criteria: (1) a score of >14.5 on the traditional Chinese version of the Eustachian Tube Dysfunction Questionnaire-7 (ETDQ-7) [18] (subjective); (2) symptoms persisting for >3 months (subjective); and (3) poor results in the inflation–deflation test or a Type B/C tympanogram (objective) [19,20,21]. All patients were refractory to at least 1 month of conservative treatment consisting of nasal corticosteroids or antihistamines. The patient demographics and baseline characteristics are shown in Table 1.

Patients aged >80 years and pregnant women were excluded to minimize the surgical risk and eliminate confounding factors. Individuals with a history of head and neck cancer, previous ear or eustachian tube surgery, or head and neck irradiation were also excluded to avoid the inclusion of variables that could affect eustachian tube function. For procedural safety and adequate endoscopic visualization, patients with severe nasal septal deviation, markedly hypertrophic (boggy) turbinates, or other unfavorable nasal anatomy were excluded from the treatment protocol.

Additional exclusion criteria included a history or presence of a tympanostomy tube, ipsilateral tympanic membrane perforation, a patulous eustachian tube, Meniere’s disease, superior semicircular canal dehiscence, and temporomandibular joint disorder. Patients with chronic rhinosinusitis, gastroesophageal reflux disease, active upper respiratory tract infections, or cystic fibrosis were also excluded. Finally, patients with structural or congenital abnormalities, such as extrinsic eustachian tube compression, cleft palate, craniofacial anomalies, or any head and neck surgery within the preceding 3 months, were excluded from the study.

### 2.3. Local Anesthesia Protocol and Surgical Procedure

To ensure a smooth, well-tolerated procedure, a standardized LA protocol was applied approximately 30 min before surgery. The patient was administered an intramuscular injection of ketorolac for analgesia. Pledgets soaked in 2% lidocaine and 1% epinephrine were then placed along the inferior nasal cavity for approximately 10 min and subsequently removed. Additional pledgets coated with lidocaine gel were applied around the pharyngeal orifice of the eustachian tube to achieve deeper topical anesthesia. Under endoscopic guidance, a balloon catheter was introduced through the nasal cavity into the cartilaginous portion of the eustachian tube. When the position had been confirmed, the balloon was inflated twice to 10 bar using distilled water, and each dilation was maintained for 2 min. All procedures were performed by a single surgeon using the LA protocol to ensure consistency and procedural safety. No adjunctive nasal or otological procedures were performed. This protocol was developed to address patient preferences for avoiding sedation and to improve the practicality of performing BET in a routine outpatient setting.

#### Eustachian Tube Function Measurements

Eustachian tube function (ETF) was evaluated using a combination of subjective and objective tests. Subjective assessments included the traditional Chinese version of the Eustachian Tube Dysfunction Questionnaire-7 (ETDQ-7) [18] and the Valsalva maneuver. The ETDQ-7 is a validated questionnaire used to quantify the severity of symptoms related to eustachian tube dysfunction. A positive Valsalva maneuver was defined as the patient’s ability to perceive a characteristic “pop” sensation in the ear during the maneuver. Objective assessments included tympanometry and an inflation–deflation (nine-step) test. Tympanometric tracings were categorized as types A, B, or C based on their shape. In the nine-step test, the maximum peak pressure difference (MPD) between the positive and negative pressure conditions was measured. In this study, MPD < 10 daPa was defined as a poor result, representing impaired eustachian tube pressure equalization [1,22,23].

### 2.4. Statistical Analysis

This prospective study was designed primarily to evaluate the feasibility and tolerability of a simplified local anesthesia protocol for BET. Based on prior clinical experience and published literature [15,16], we anticipated a procedural completion rate of approximately 90–95% and a pain acceptability proportion (defined as VAS ≤ 5) of approximately 80%. The sample size was therefore determined to allow for the estimation of these feasibility outcomes with clinically meaningful precision. With 50 participants, the expected half-width of the 95% confidence interval was approximately ±6–8% for the procedural completion rate and ±11% for pain acceptability, which was considered sufficient for feasibility assessment. Continuous variables are presented as means ± standard deviation (SD), and categorical variables as counts and percentages. Procedural pain scores (VAS), blood pressure, procedural completion and tolerability rates, and willingness to undergo local anesthesia again were analyzed on a per-patient (per-procedure) basis.

Ear-level outcomes, including ETDQ-7 scores, tympanogram type, and Valsalva maneuver results, were recorded separately for each treated ear. Because bilateral ears from the same patient are not independent, ear-level analyses were performed using generalized estimating equations (GEE) with patient identification as the clustering variable, an exchangeable working correlation structure, and robust standard errors. Continuous ear-level outcomes were modeled assuming a normal distribution with an identity link, whereas binary outcomes were modeled using a binomial distribution with a logit link. Based on the baseline ETDQ-7 standard deviation (7.4), the minimal clinically important difference (MCID) was estimated to be approximately 3.7. All statistical tests were two-tailed, with statistical significance defined as *p* < 0.05. Analyses were conducted using SPSS software (version 20.0; IBM Corp., Armonk, NY, USA).

## 3. Results

### 3.1. ETDQ-7 Improvement and Normalization

The mean ETDQ-7 score improved significantly from 24.9 ± 7.4 preoperatively to 11.9 ± 5.4 at 3 months (Δ = 12.9 ± 7.6). When ear-level correlation between paired ears was accounted for using generalized estimating equations (GEE), postoperative ETDQ-7 scores remained significantly lower than baseline (β = −12.94, 95% CI −14.93 to −10.94; *p* < 0.001). A clinically meaningful improvement exceeding the minimal clinically important difference (MCID > 3.7) was achieved in 58/64 ears (90.6%). ETDQ-7 normalization (≤14.5) was observed in 48/64 ears (75.0%).

### 3.2. Positive Valsalva Maneuver

The ability to successfully perform the Valsalva maneuver improved markedly after BET. The proportion of ears demonstrating a positive Valsalva maneuver increased from 25/64 (39.1%) preoperatively to 49/64 (76.6%) postoperatively. In logistic GEE analysis accounting for within-patient correlation between paired ears, the odds of a positive Valsalva maneuver were significantly higher after BET (odds ratio OR = 5.06, 95% CI 2.39–10.74; *p* < 0.001).

### 3.3. Tympanogram

The proportion of type A tympanograms increased from 41/64 (64.1%) preoperatively to 54/64 (84.4%) postoperatively (OR = 2.99, 95% CI 1.66–5.39; *p* < 0.001). Correspondingly, type B and C tympanograms decreased from 25.0% and 10.9% to 9.4% and 6.3%, respectively, indicating improved middle ear ventilation after BET. The postoperative results are summarized in Table 2.

### 3.4. Pain Scores During the Procedure

The mean pain scores on the VAS were 3.5 ± 1.5 during balloon insertion, 2.1 ± 1.4 during inflation and maintenance of the pressure, and 0.6 ± 0.6 1 min after BET (Table 3). All procedures were successfully completed under LA, and 96% of the patients reported that they would choose LA again, indicating high tolerability and overall satisfaction.

### 3.5. Blood Pressure During the Procedure

As shown in Table 3, the mean systolic blood pressure increased slightly from 134.1 ± 21.4 mmHg preoperatively to 148.3 ± 21.7 mmHg intraoperatively. The mean diastolic blood pressure also increased mildly from 78.6 ± 12.0 mmHg to 86.2 ± 11.0 mmHg during the procedure. This mild intraoperative increase in blood pressure is likely attributable to sympathetic activation or pain from procedural stimulation but remained within a safe physiological range.

## 4. Discussion

### 4.1. Feasibility and Efficacy of LA-BET

For surgeons, the ability to perform BET efficiently in the clinic without compromising patient comfort is extremely convenient. This study demonstrated that BET performed under LA could be completed safely and effectively with a 100% procedural success and a 96% willingness of patients to undergo the procedure again. Adequate pain control remains the primary challenge of LA-BET; however, the intraoperative discomfort was mild, and no major complications were observed. The transient increase in blood pressure further supports the overall tolerability of the procedure. Significant postoperative improvements in the ETDQ-7 scores, tympanogram types, and Valsalva maneuver test results confirmed that LA-BET is feasible and clinically effective. Our simplified protocol of intramuscular ketorolac premedication combined with topical lidocaine gel anesthesia and slow balloon inflation provided reliable analgesia while remaining technically simple and efficient. In many cases, patients reported that the simplicity of this procedure made them more willing to proceed with the treatment rather than delay care.

### 4.2. Comparison with Previous Studies

Compared with those in previous reports (Table 4), the anesthesia protocol used in this study was remarkably simplified, while maintaining effective analgesia and procedural success rates. Luukkainen et al. (2017) used cocaine pledgets with intravenous fentanyl or midazolam [13], whereas Dean (2019) and Toivonen et al. (2022) used high-concentration tetracaine/lidocaine anesthesia, often combined with diazepam premedication, which occasionally causes postoperative drowsiness [14,16]. In contrast, Møller (2025) incorporated a sphenopalatine ganglion (SPG) block [17], effectively reducing pain from a mean VAS of 6.7 to 1.4 but increasing the risk of mucosal bleeding extending toward the eustachian tube orifice, potentially impairing visualization for less-experienced operators. Previous studies have generally reported mean intraoperative pain scores of approximately 4–6 [13]. This is consistent with our findings when using a simplified ketorolac + lidocaine protocol (VAS ≈ 3). Adequate analgesia was achieved without oral sedatives or nerve blocks, and all procedures were successfully completed under LA [17,24,25].

Differences in balloon design may also influence pain perception; wider devices, such as the Acclarent Aera (±6 mm), can produce greater mucosal tension and pain than narrower systems, such as the Spiggle & Theis TubaVent (3.28 mm) [13]. In the present study, we used a 20 mm × 3.28 mm balloon, which was inflated slowly to 10 bars for 2 min, providing sufficient dilation with minimal discomfort. As in the study by Møller [17], who inflated the balloon twice, our two-cycle approach maintained tolerable pain throughout the procedure. Patients often report pain peaks during the initial insertion, which diminish during the second inflation, likely due to progressive luminal expansion and reduced mucosal tension. Overall, the combination of ketorolac premedication, topical lidocaine gel, and slow inflation achieved an optimal balance among analgesic efficacy, procedural simplicity, and patient safety.

### 4.3. Mechanistic Insight

Pain perception during BET is likely mediated by mechanical stimulation of the mucosa in the cartilaginous portion of the eustachian tube and adjacent nasopharyngeal region. The nasopharyngeal mucosa, including the area surrounding the pharyngeal orifice of the eustachian tube, receives sensory innervation from the pharyngeal branch of the maxillary division of the trigeminal nerve (V2), with additional neural connections involving the sphenopalatine ganglion [26,27]. Targeting these neural pathways, such as through the SPG block, substantially reduces procedural pain [17]. Mechanical stretching during balloon inflation increases wall tension within these mechanosensitive tissues, activating nociceptive pathways, and producing discomfort or transient otalgia. Smith et al. demonstrated that balloon dilation can cause mucosal tears, submucosal disruption, and microfractures within the cartilaginous portion of the eustachian tube [8].

In our protocol, topical lidocaine gel was applied directly around the eustachian tube orifice to desensitize the local mucosa, whereas intramuscular ketorolac provided systemic analgesia through peripheral prostaglandin inhibition. Together, these mechanisms provide effective pain control without the need for sedatives or nerve blocks and offer a simple solution suitable for outpatient BET.

### 4.4. Clinical Implications

From a clinical standpoint, LA-BET can be safely and efficiently performed in an outpatient setting, eliminating the need for GA and reducing the procedural time and cost. Across all published LA-BET studies (Table 4), procedural completion rates exceeded 90%, and 96–100% of patients who were specifically surveyed expressed their willingness to undergo the procedure again, reflecting high satisfaction. Given its short duration, low cost, minimal invasiveness, and favorable safety profile, LA-BET may serve as the preferred first-line intervention for older adult or medically fragile patients who are unsuitable for GA.

### 4.5. Limitations & Future Directions

Several limitations of the present study should be acknowledged. First, the absence of a control group undergoing BET under GA precludes direct comparative conclusions regarding efficacy between anesthesia modalities. Accordingly, the findings should be interpreted primarily in the context of procedural feasibility, safety, and patient tolerability, rather than comparative efficacy. Second, this was a single-center study with a limited sample size, which may restrict the generalizability of the findings. Although adequate for feasibility assessment, larger multicenter studies are warranted to further validate the clinical applicability.

Third, the follow-up period was limited to 3 months, precluding assessment of long-term durability of symptom relief and functional outcomes. Longer-term follow-up studies are needed to determine the sustainability of clinical benefits after LA-BET. Fourth, postoperative pain assessment focused on intraoperative and immediate postoperative pain, and delayed pain beyond the early postoperative period was not systematically evaluated. While delayed pain is generally minimal following in-office BET, future studies incorporating extended pain assessments may provide a more comprehensive evaluation of patient comfort and recovery.

Finally, the present analysis did not compare different balloon sizes or inflation durations. Future studies exploring these procedural variables may help identify the optimal balance between pain control, procedural tolerability, and therapeutic efficacy in patients undergoing LA-BET.

## 5. Conclusions

LA-BET, using a simplified protocol with ketorolac and topical lidocaine gel, is feasible, safe, and effective. The patients experienced significant symptomatic and functional improvements, with minimal discomfort and without major complications. Given its simplicity and good tolerability, LA-BET is a practical option for the outpatient management of OETD.

## Figures and Tables

**Table 1:** Patient demographics and baseline characteristics (N = 50 patients, 64 ears).

Variables	Before LA-BET
Age (years)	51.0 ± 13.5
Sex (female: male)	22:28
Asthma, n (%)	4 (8%)
Allergic rhinitis, n (%)	9 (18%)
Bilateral, n (%)	14 (28%)
Unilateral, n (%)	36 (72%)
Eustachian tube function	
ETDQ-7	24.9 ± 7.1
E-tube mucosa inflammation scale	2.34 ± 0.70
Valsalva maneuver, n (%)	25/64 (39.1%)
Type A tympanogram, n (%)	41/64 (64.1%)
Type B tympanogram, n (%)	16/64 (25.0%)
Type C tympanogram, n (%)	7/64 (10.9%)

LA-BET, local anesthesia-balloon eustachian tuboplasty; ETDQ-7, Eustachian Tube Dysfunction Questionnaire-7.

**Table 2:** Postoperative results (N = 64 ears).

Variables	After LA-BET
ETDQ-7	11.9 ± 5.4
ETDQ-7 improvement	12.9 ± 7.6
ETDQ-7 change at least MCID (3.7), n (%)	58 (90.6%)
ETDQ-7 normalization (≤14.5), n (%)	48 (75.0%)
Positive Valsalva maneuver, n (%)	49 (76.6%)
Type A tympanogram, n (%)	54 (84.4%)
Type B tympanogram, n (%)	6 (9.4%)
Type C tympanogram, n (%)	4 (6.3%)

BET, balloon eustachian tuboplasty; ETDQ-7, Eustachian Tube Dysfunction Questionnaire-7; MCID, minimal clinically important difference.

**Table 3:** Pain and perioperative blood pressure (N = 50).

Pain Score (VAS)	Mean ± SD
Balloon catheter insertion	3.5 ± 1.5
During pressure maintenance at 10 bars	2.1 ± 1.4
1 min postoperatively	0.6 ± 0.6
Systolic blood pressure (mm Hg)	
Preoperative	134.1 ± 21.4
Intraoperative	148.3 ± 21.7
Diastolic blood pressure (mm Hg)	
Preoperative	78.6 ± 12.0
Intraoperative	86.2 ± 11.0

VAS, visual analogue scale; SD, standard deviation.

**Table 4:** Summary of published studies of LA-BET: protocols and outcomes.

Study (Year)	Number of Patients (Ears)	LA Protocol/Sedation	Intraoperative VAS	Outcome Summary
Luukkainen (2017) [13]	18 (27)	Cocaine + adrenaline pledgets → lidocaine–prilocaine cream; IV fentanyl ± midazolam	5.0 ± 0.7	Completion: 100%; 92% would repeat; feasible under MAC
Meyer et al. (2018) [24]	38	Topical lidocaine + epinephrine spray ± injection; ± oral sedative (diazepam)	4.1 ± 2.8	Completion: 100%; Type A tympanogram: 88% Valsalva maneuver (+): 66% ETDQ-7: 4.6 → 2.1
Dean (2019) [14]	33 (43)	Diazepam premed →oxymetazoline→ 7% tetracaine/lidocaine cream to ET orifice	NR	Completion: 94%; Type A tympanogram: 87%
Chen et al. (2020) [15]	25 (32)	1% lidocaine + epinephrine pads → 0.5 mL lidocaine cream; no sedation	Insert: 5.4 ± 1.7 Inflation: 6.1 ± 1.0 Maintain: 4.9 ± 2.2	Completion: 96%; 96% would repeat; ETDQ-7 normalization: 72%; Type A tympanogram: 95%; Valsalva maneuver (+): 96% no efficacy difference vs. GA
Toivonen et al. (2022) [16]	58 (107)	Refined topical anesthesia (oxymetazoline + 7% tetracaine/lidocaine) diazepam premed optional	NR	Completion: 99%; Type A tympanogram: 88% outcomes comparable to GA
Ungar et al. (2024) [25]	8 (12)	Oxymetazoline spray → 2% tetracaine cottonoids (15 min); no sedation	Mild discomfort	Completion: 100%; 100% would repeat; ETDQ-7: 4.4→2.3 Type A tympanogram:67%; Valsalva maneuver (+): 75%
Møller (2025) [17]	511 (1001)	Protocol 1: tetracaine/phenylephrine + xylocaine spray → Protocol 2: + lidocaine/prilocaine gel + oxazepam 15 mg → Protocol 3: + SPG block (bupivacaine + adrenaline 2–3 mL/side)	Protocol 1 = 6.7 Protocol 2 = 3.5 Protocol 3 = 1.4	Completion: 100% 96% would repeat; Type A tympanogram:69% Valsalva maneuver (+): 76% ETDQ-7 normalization: 82%; minor bleeding < 2%
Hsieh et al. (2025) Present study	50 (64)	Ketorolac→ 2% lidocaine + 1% adrenaline pledgets → lidocaine gel	Insert: 3.5 Maintain: 2.1	Completion: 100% 96% would repeat; Type A tympanogram: 84.4% Valsalva maneuver (+): 76.6% ETDQ-7 normalization: 75%

ETDQ-7, Eustachian Tube Dysfunction Questionnaire-7; SPG, sphenopalatine ganglion; VAS, visual analog scale; MAC, monitored anesthesia care; GA, general anesthesia. ETDQ-7 normalization = postoperative total score < 14.5.

## Data Availability

Data are contained within the article.

## Abbreviations

The following abbreviations are used in this manuscript:

BET	Balloon eustachian tuboplasty
LA	Local anesthesia
LA-BET	Balloon eustachian tuboplasty under local anesthesia
OETD	Obstructive eustachian tube dysfunction
ETDQ-7	Eustachian Tube Dysfunction Questionnaire-7
ETD	Eustachian tube dysfunction
GA	General anesthesia
VAS	Visual analogue scale
SD	Standard deviation
MCID	Minimal clinically important difference
ETF	Eustachian tube function
